# Behavioral inhibition and dual mechanisms of anxiety risk: Disentangling neural correlates of proactive and reactive control

**DOI:** 10.1002/jcv2.12022

**Published:** 2021-07-02

**Authors:** Emilio A. Valadez, Sonya V. Troller‐Renfree, George A. Buzzell, Heather A. Henderson, Andrea Chronis‐Tuscano, Daniel S. Pine, Nathan A. Fox

**Affiliations:** ^1^ Department of Human Development and Quantitative Methodology University of Maryland College Park Maryland USA; ^2^ Department of Biobehavioral Sciences Teachers College Columbia University New York New York USA; ^3^ Department of Psychology Florida International University Miami Florida USA; ^4^ Department of Psychology University of Waterloo Waterloo Ontario Canada; ^5^ Department of Psychology University of Maryland College Park Maryland USA; ^6^ Emotion and Development Branch National Institute of Mental Health Intramural Research Program National Institute of Mental Health Bethesda Maryland USA

**Keywords:** adolescence, anxiety, behavioral inhibition, cognitive control, EEG

## Abstract

**Background:**

Behavioral inhibition (BI) is a temperament style characterized by heightened reactivity and negative affect in response to novel people and situations, and it predicts anxiety problems later in life. However, not all BI children develop anxiety problems, and mounting evidence suggests that how one manages their cognitive resources (cognitive control) influences anxiety risk. The present study tests whether more (proactive control) or less (reactive control) planful cognitive strategies moderate relations between early BI and later anxiety.

**Methods:**

Participants included 112 adolescents (55% female; *M*_*age*_ = 15.4 years) whose temperament was assessed during toddlerhood. In adolescence, participants completed an AX Continuous Performance Test while electroencephalography was recorded to disentangle neural activity related to proactive (cue‐locked P3b) and reactive (probe‐locked N2) control.

**Results:**

Greater BI was associated with greater total anxiety scores only among adolescents with smaller ΔP3bs and larger ΔN2s—a pattern consistent with decreased reliance on proactive strategies and increased reliance on reactive strategies. Additionally, a larger ΔP3b was associated with greater total anxiety scores; however, this effect was largely explained by the fact that females tended to have larger ΔP3bs and greater anxiety than males.

**Conclusions:**

Early BI relates to risk for later anxiety specifically among adolescents who rely less on proactive strategies and more on reactive control strategies. Thus, cognitive control strategy moderates the association between developmental context (i.e., temperament) and later anxiety. The present study is the first to characterize how proactive and reactive control uniquely relate to pathways toward anxiety risk.

## INTRODUCTION

Behavioral inhibition (BI) is a temperament style characterized by heightened reactivity and negative affect in response to novel people and situations (Kagan et al., 1984). Although there is some debate regarding whether BI should be conceptualized as categorical or continuous, it is typically quantified as a continuous score based on coded laboratory observation, parental report, or a combination thereof (Clauss & Blackford, 2012). High BI predicts later‐life anxiety problems (Fox et al., 2005; Fox & Pine, 2012; Schwartz et al., 1999), especially when high BI is stable throughout infancy and early childhood (Chronis‐Tuscano et al., 2009). Nevertheless, 30%–60% of toddlers with high BI do not go on to meet criteria for an anxiety disorder during childhood or adolescence (Clauss & Blackford, 2012; Gladstone et al., 2005). Thus, identifying factors that moderate the relation between BI and anxiety remains a key issue for prevention and intervention.

**Key points**

- Behaviorally inhibited (BI) temperament is a strong predictor of anxiety problems later in life, but this association is moderated by cognitive control factors.
- By separating proactive and reactive control processes using electroencephalography, the present study is the first to characterize how proactive and reactive control uniquely relate to pathways toward anxiety risk.
- Findings suggest that BI relates to risk for anxiety specifically among adolescents who rely less on proactive strategies and more on reactive control strategies.
- Reliance on proactive control strategies was also independently related to anxiety risk, but this effect was largely explained by the fact that females tended to use more proactive strategies and had greater anxiety than males.

The BI literature has examined many potential moderators of the BI‐anxiety association, including parent characteristics (e.g., maternal anxiety and negativity) and parent‐child relationship factors (e.g., parental overinvolvement and mother‐child attachment; Degnan et al., 2010; Hudson & Dodd, 2012; Kiel & Buss, 2011; Lewis‐Morrarty et al., 2012; Rubin et al., 2002). Indeed, interventions targeting these mechanisms have shown promise (Chronis‐Tuscano et al., 2015; Rapee et al., 2005). Yet, children's own self‐regulatory skills, particularly their cognitive control skills—skills involved in monitoring and adapting behavior in accordance with goals—have most consistently been shown to modulate anxiety risk for children with BI (Fox et al., 2021; Henderson, 2010; Lamm et al., 2014; McDermott et al., 2009; Smith et al., 2019; Troller‐Renfree, Buzzell, Bowers, et al., 2019; Troller‐Renfree, Buzzell, Pine, et al., 2019).

The dual‐mechanisms of control (DMC) theory (Braver, 2012) differentiates two temporally distinct and complementary, yet largely independent, strategies of cognitive control: proactive and reactive. Proactive control involves early selection and maintenance of goal‐relevant information over time, whereas reactive control involves in‐the‐moment recruitment of resources, often in response to conflict. A key distinction between proactive and reactive control involves the timing during which processing occurs. That is, proactive control is employed *early* in time (commonly in a preparatory fashion) and may involve adapting to and maintaining task set changes appropriate to task context (e.g., changes in task rules, shifting to a new activity, etc.). However, reactive control is employed *later* in time, usually after a stimulus or other event of interest has occurred (Braver, 2012). For example, proactive control may be involved in selecting and maintaining a child's overarching goal of playing a game with peers; on the other hand, seeing a peer's angry face may trigger reactive control processes that disrupt proactive goal maintenance, shifting the attention set away from the game and onto the peer's expression.

One emerging view of BI's neurophysiological profile recognizes the temperament's association with heightened detection of salient stimuli (e.g., threatening faces; for a recent review, see Fox et al., 2021). However, this view also notes that some children with BI learn to regulate their responses to novelty, unfamiliarity, or other salient cues over time via increased proactive control (Buzzell et al., 2018; Fox et al., 2021; Henderson et al., 2015; Henderson & Wilson, 2017). This increased proactive control helps the child recover their goal‐oriented attention (e.g., refocusing attention back toward playing the game) and reduces the length of time that attention is shifted toward the salient or unexpected stimulus (e.g., the peer's potentially threatening facial expression). This increase in proactive control thereby reduces the risk for anxiety. Of note, we developed this framework specifically to help understand the BI‐anxiety association. Nevertheless, the framework is in part rooted in a long history of work finding that facets of cognitive control moderate the relation between stable individual characteristics (e.g., personality traits such as negative affectivity or neuroticism) and pathological anxiety (e.g., see Lonigan et al., 2004). Our framework also aligns with attentional control theory (Eysenck et al., 2007; Eysenck & Derakshan, 2011). This theory highlights a large body of literature connecting anxiety to poor proactive task switching and lower efficiency of reactive inhibitory control (i.e., greater inhibitory control with no corresponding increases in overall performance; for a meta‐analysis, see Shi et al., 2019).

One of the few available tasks used to measure the dual mechanisms of control is the AX continuous performance test (AX‐CPT; Barch et al., 1997; Cohen et al., 1999). The AX‐CPT presents a continuous series of letter pairs (i.e., a cue letter followed by a probe letter) dissociated into four trial types (AX, AY, BX, and BY), each reflecting a different combination of the cue (e.g., the letters “A” or “B”) and probe (e.g., the letters “X” or “Y”). Participants are instructed to press a button (e.g., “1”) following every cue and following most types of probes. They are also instructed that whenever they see an “A” (target cue) followed by an “X” (target probe), they are to press a different button (e.g., “4”). The AX‐CPT enables measurement of both proactive and reactive control through weighting the probability of trials differently (i.e., some trial types are more common than others) and the use of contextual cues that inform the response to the upcoming probe.

Control processes are typically measured by comparing accuracy and/or reaction time (RT) across different trial types. Participants using a more proactive strategy (i.e., those paying more attention to the cue) are expected to experience higher conflict (i.e., slower RT, more errors) on AY trials. Conversely, those using a more reactive strategy (i.e., those paying more attention to the probe) are expected to experience higher conflict on BX trials. As a result, behavioral studies have largely relied on the difference between AY and BX trials to measure proactive and reactive control (Braver et al., 2009; Yang et al., 2018). However, individual differences in this AY‐BX contrast are difficult to interpret because they could be driven by differences in reactive control, proactive control, or both. This is problematic because proactive and reactive control are thought to be relatively independent processes, not two ends of the same continuum (Braver, 2012; Gonthier, Braver, et al., 2016). Another commonly used behavioral index that does *not* conflate proactive and reactive control is *d*' context. *d*' context is based on signal detection theory and involves comparing hit rate on AX trials versus false alarm rate on BX trials. As such, it measures the ability to discriminate between target and nontarget trials as a function of the cue, thus providing a relatively pure measure of proactive control (Cohen et al., 1999).

A recent longitudinal study examined whether *d*' context scores moderated the relation between BI and anxiety. This study found that 13‐year‐old children with a history of high BI during toddlerhood tended to use a relatively less proactive strategy than children without such history, as indicated by lower *d*' context scores on the AX‐CPT (Troller‐Renfree, Buzzell, Pine, et al., 2019). Moreover, *d*' context scores moderated the relations between BI and parent‐reported anxiety such that children with high BI who used a less proactive strategy (i.e., lower *d*' context scores) had greater total anxiety at age 13 than children with BI who used a more proactive strategy (i.e., higher *d*' context scores; Troller‐Renfree, Buzzell, Pine, et al., 2019). Additional support exists for the idea that children high in both BI and anxiety may utilize less proactive (and more reactive) strategies. This support comes from studies of this same cohort of children, who also showed increased performance on tasks necessitating reactive conflict detection, such as a Go/Nogo task (Troller‐Renfree, Buzzell, Bowers, et al., 2019) and Day‐Night and Grass‐Snow Stroop tasks (White et al., 2011). These children also demonstrated decreased performance on a task necessitating proactive task‐switching (the Dimensional Change Card Sort; White et al., 2011). Finally, at least one study from an independent cohort showed that BI was associated with greater anxiety specifically among children with greater reactive inhibitory control on a Go/Nogo task (Thorell et al., 2004).

Yet, more confirmatory evidence is lent by studies of brain function. These studies find increased neural recruitment in high‐conflict scenarios by children high in both BI and anxiety, as measured via event‐related potentials (ERPs) from electroencephalography (EEG). Youth from the same cohort examined by Troller‐Renfree, Buzzell, Pine, et al. (2019) were studied in one such report. This study found that children who were high in both BI and anxiety had larger N2 responses to conflict on a Go/Nogo task (likely indicating greater reflexive attention toward conflict—an example of reactive control; Lamm et al., 2014), with another study showing that among children with BI, larger error‐related negativity (ERN) responses to errors during a Flanker task (likely indicating greater reflexive attention toward errors—another example of reactive control) prospectively predicted increased anxiety symptoms two years later (Lahat et al., 2014 but see also Buzzell et al., 2017). Again, consistent findings have emerged from studies examining independent cohorts. These studies, too, have found youth high in both BI and anxiety to have larger N2 responses to conflict (Henderson, 2010) and larger ERNs following errors (McDermott et al., 2009). Yet another study found children high in both also have larger P3 responses to novel auditory tones, which may indicate greater reactivity to surprising stimuli (Reeb‐Sutherland et al., 2009). The bulk of ERP evidence from multiple cohorts (including this sample) suggests that children with BI who engage in more reactive control may be at greater risk for anxiety difficulties than children with BI who use a less reactive‐like control strategy.

Despite an extant literature focusing on neural measures of reactive control, no studies examining BI to date have characterized a neural measure of proactive strategy use. This is important since proactive and reactive control processes are thought to be relatively independent (Braver, 2012; Gonthier, Braver, et al., 2016). The lack of such work leaves it unclear whether the association between BI and anxiety depends on the level of reactive control (independent of proactive control), on the level of proactive control (independent of reactive control), or the interplay between the two.

Functional magnetic resonance imaging generally lacks the temporal resolution to disentangle proactive‐ and reactive‐control‐related brain activity. EEG studies of the AX‐CPT, in contrast, have been able to identify neural components that uniquely map onto proactive and reactive processes. The cue‐locked P3b component of the ERP is a positive voltage deflection maximal at centro‐parietal electrode sites typically between 350 and 450 ms following cue presentation (but prior to probe presentation; Tekok‐Kilic et al., 2001). It has been shown to index the updating of working memory prior to probe presentation, a preparatory and therefore proactive process, following changes in context in both adults (van Wouwe et al., 2011) and children (Troller‐Renfree et al., 2020). Studies consistently find larger (more positive) cue‐locked P3b amplitude following B cues than following A cues, especially among individuals using more proactive‐like strategies. This could be because B cues are both more rare (i.e., they signal a shift from the task's more common A‐cue context) and more informative (i.e., they eliminate all uncertainty about what the probe response should be) than A cues. The B‐cue minus A‐cue P3b amplitude difference was also shown to mediate the relation between children's working memory abilities and their preference for a more proactive (rather than reactive) behavioral strategy during the AX‐CPT (Troller‐Renfree et al., 2020). A larger B‐A cue‐locked P3b difference score, therefore, likely indicates a cognitive control strategy characterized by high proactive strategy use.

The probe‐locked N2 ERP component is a negative voltage deflection maximal at fronto‐central electrode sites typically between 150 and 300 ms following probe presentation (Lamm et al., 2013; Van Veen & Carter, 2002; van Wouwe et al., 2011). The N2 is generally considered to index reactive conflict detection and is larger (more negative) following less frequent stimuli, conflicting stimuli, and during inhibition of a prepotent response (Cavanagh & Frank, 2014; Van Veen & Carter, 2002). Mirroring behavioral studies, ERP studies of the AX‐CPT have generally measured the N2 as a difference score contrasting AY and BX (Troller‐Renfree, 2018; van Wouwe et al., 2011). However, as noted earlier, this AY‐BX contrast can be modulated by differences in proactive control, reactive control, or both—limiting interpretability. Critically, EEG enables dissociation of cue‐ and probe‐related processing, thereby reducing reliance on such confounded trial contrasts. For example, because AX and BX trials share the same probe identity, the difference in probe‐locked N2 amplitude between the two captures activity related to reactive strategy that is not confounded by the probe identity. This also allows a direct comparison to the cue‐locked P3b. That is, whereas a larger cue‐locked B‐A P3b difference score indicates the extent to which the cue identity is being processed *before the probe* (suggesting the use of proactive control), a larger probe‐locked AX‐BX N2 difference score indicates the extent to which the cue identity is being processed *after the probe* (suggesting the use of reactive control). Thus, these separate cue‐locked and probe‐locked ERP difference scores each provide unique information about an individual's cognitive control strategy.

As noted earlier, no BI study has examined neural measures of both proactive and reactive control. This, coupled with the fact that proactive and reactive control cannot be separated based on established behavioral metrics alone, leaves it unknown whether the relation between BI and anxiety depends primarily on proactive control, reactive control, or on the interaction between the two. To answer this question, participants enrolled as part of a longitudinal study were assessed for BI during toddlerhood and completed an AX‐CPT task modified for EEG compatibility at age 15 years. Importantly, the present study utilized separate neural measures of proactive and reactive control in order to test whether behavioral findings from an earlier time point of this longitudinal study (Troller‐Renfree, Buzzell, Pine, et al., 2019) were driven mainly by proactive control, by reactive control, or by their interaction. Past behavioral findings connect BI to anxiety among youth who employ relatively less proactive control (Troller‐Renfree, Buzzell, Pine, et al., 2019; White et al., 2011); ERP findings connect BI to anxiety among youth who employ relatively more reactive control (Henderson, 2010; Lahat et al., 2014; Lamm et al., 2014; McDermott et al., 2009; Reeb‐Sutherland et al., 2009). Thus, in this new 15‐year EEG assessment, we hypothesized that youth anxiety would be associated with a three‐way interaction involving BI, proactive control, and reactive control. That is, we predicted that BI would be associated with greater anxiety, specifically among participants using a cognitive control strategy characterized by low proactive control (i.e., smaller B‐A cue‐locked P3b difference scores) and high reactive control (i.e., larger AX‐BX probe‐locked N2 difference scores).

## MATERIALS AND METHODS

### Participants

Participants included 167 adolescents (with 112 included in primary analyses—see below) aged 15‐17 years (*M* = 15.4 years, SD = 0.6; 56% female) who were administered an AX‐CPT task during EEG recording as part of a longitudinal study examining the relations between infant temperament and the emergence of anxiety. Participants were 17% African American, 6% Hispanic/Latino, 3% Asian, 71% Caucasian, and 3% “Other,” as identified by their parents. This study's recruitment strategy and screening methods have been described in detail elsewhere (Calkins et al., 1996; Fox et al., 2001; Hane et al., 2008; see Appendix S1; Walker et al., 2014). The attrition rate from infancy (*n* = 291) to the 15‐year AX‐CPT assessment (*n* = 167) was 42.6%. Chi‐squared and *t*‐tests revealed no significant differences between those who did versus did not participate in the 15‐year AX‐CPT assessment in terms of race/ethnicity, maternal education level, sex, or BI (all *p*s > 0.34).

### Behavioral inhibition

BI was assessed at ages 24 and 36 months and included a combination of behavioral coding of laboratory assessments (Calkins et al., 1996; Fox et al., 2001) and maternal report of social fear (see Appendix S1).

### AX continuous performance task

To measure distinct neural indices of proactive and reactive control, participants completed an AX‐CPT (Barch et al., 1997; Braver, 2012; Cohen et al., 1999) that was modified for simultaneous EEG recording. Consistent with past ERP studies involving the AX‐CPT, the traditional 70%/10%/10%/10% trial breakdown (reflecting AX/AY/BX/BY trials) was modified to 55%/15%/15%/15%. This change was made to achieve adequate ERP signal‐to‐noise ratio for each trial type without excessively extending task duration (Lamm et al., 2013; Troller‐Renfree, 2018). The task included 319 trials (175/48/48/48) presented in random order across four blocks. Importantly, as in behavioral versions of the task, AX trials were by far the most frequent; thus, behavioral predictions remain the same as in most past studies using the AX‐CPT. That is, individuals using predominantly proactive strategies were predicted to commit more errors on AY trials and fewer errors on BX trials compared to those using predominantly reactive strategies (Braver et al., 2009; Cohen et al., 1999; Gonthier, Macnamara, et al., 2016). See Appendix S1 for additional detail regarding stimuli, cleaning of behavior data, and calculation of *d'* context.

### Screen for child anxiety related emotional disorders

Each participant and their parent completed the revised version of the screen for child anxiety related emotional disorders (SCARED; Monga et al., 2000) at the 15‐year assessment. The parent and child versions of the SCARED included 41 items presented on a 3‐point Likert scale (0 = never/hardly ever true, 1 = sometimes/somewhat true, 2 = very/often true). Total anxiety scores were the primary outcome of interest (parent version: *α* = 0.93; child version: *α* = 0.92). To combine information from multiple informants while also accounting for differences in how parents and children rate anxiety symptoms, total anxiety scores were computed separately for parent and child, Z‐transformed, and then averaged together to form an anxiety composite score for analyses. This Z‐transformed composite score has been shown to relate to a wider variety of naturalistically observed anxious behaviors than either parent‐ or child‐report alone (Bowers et al., 2020). For separate regression results involving parent‐reported or child‐reported anxiety, see Tables S1–S4. Although not included in analyses, participants were also administered a semi‐structured interview assessing past and current psychopathology. Descriptive data regarding mood and anxiety diagnoses are presented in Appendix S1.

### Electrophysiological recording, pre‐processing, and analysis

Continuous EEG was recorded using a 128‐channel Geodesic Sensor Net (Electrical Geodesics, Inc.) and sampled at 250 Hz. Before data collection, all electrode impedances were reduced to <50 kΩ. During data collection, electrodes were referenced to electrode Cz. See Appendix S1 for additional pre‐processing details. A Laplacian transform was applied to convert epoch data from μV to V/m^2^ (i.e., current source density), thus improving spatial resolution (Tenke & Kayser, 2012). All ERPs were aligned to a baseline of −200 to 0 ms with respect to stimulus onset. Each ERP component was scored by first identifying the positive (P3b) or negative (N2) peak within the scoring window for the given component (see time windows below) and then averaging the amplitudes from 40 ms (i.e., 10 samples) pre‐peak to 40 ms post‐peak. This adaptive mean scoring approach was used because it is more robust to potential individual differences in peak latency than averaging across the entire scoring window, while still representing an efficient estimation of the true ERP amplitude (Clayson et al., 2013). Sensors for the centroparietal (P3b) and frontocentral (N2) regions‐of‐interest were selected based on the topography of the grand average waveforms. Scoring time windows (described below) corresponded to the latencies between which the grand average waveforms exceeded approximately half the peak‐to‐peak amplitude (with respect to the preceding and following peaks). This data‐driven scoring approach was used in light of findings of age‐related differences in ERP latency (Boutet et al., 2021; Gavin et al., 2019) and has been used in past ERP studies of the AX‐CPT (van Wouwe et al., 2011).

For both ERP components, subtraction‐based difference scores were used rather than residualized difference scores because proactive and reactive strategy use each were operationalized as the extent to which participants differentiated between A versus B cues and AX versus BX probes, respectively. If taking a residualized approach, any such ERP amplitude differences would have been largely regressed out due to sharing variance across both single‐condition ERPs. In other words, residualized scores do not allow for comparisons across conditions. Furthermore, recent work suggests that subtraction‐based and residualized ERP difference scores have similar internal consistency and that subtraction‐based scores are likely preferable due to their greater parsimony and interpretability (Clayson et al., 2021). Nevertheless, for comparison to results from subtraction‐based ERP difference scores, results of a regression model using residualized scores are presented in Table S5.

#### P3b

The cue‐locked P3b was used as the measure of proactive control. The search space for the P3b peak amplitude was limited to a time window of 430 to 680 ms post‐cue after averaging across centroparietal sensor sites (E31, E54, E55, E79, and E80). Analyses focused on a difference score reflecting B minus and A trials (ΔP3b), with a larger (more positive) difference indicating more proactive control use (Troller‐Renfree et al., 2020; van Wouwe et al., 2011). Reliability analyses revealed that a minimum of 10 trials for A cues and 6 trials for B cues were needed to achieve acceptable reliability (see Appendix S1 and Figure S1).

#### N2

The probe‐locked N2 was used as the measure of reactive control. The search space for the N2 peak amplitude was limited to a time window of 260 to 350 ms post‐probe after averaging across frontocentral sensor sites (E5, E6, E7, E12, E13, E106, and E112). Analyses of the probe‐locked N2 focused on a difference score reflecting AX minus BX trials (ΔN2), with a larger (more negative) difference indicating more reactive control use. Reliability analyses revealed that a minimum of 12 trials for AX probes and 18 trials for BX probes were needed to achieve acceptable reliability (see Appendix S1 and Figure S1).

### Data analytic strategy

To assess whether proactive and reactive control moderated the relations between BI and anxiety, two linear regression models were tested in R (version 3.6.2) with the function “lm”. The outcome variable in both models was parent‐ and child‐reported total anxiety from the SCARED, which, as noted earlier, were Z‐transformed and then averaged together to create a composite total anxiety score. The first model was included as a partial replication of our previous investigation (Troller‐Renfree, Buzzell, Pine, et al., 2019). The model included BI, *d'* context (as a behavioral measure of proactive vs. reactive control), and their interaction as predictors. In the second model, predictors included BI, ΔP3b, ΔN2, and their two‐ and three‐way interactions. For similar ERP regression models predicting specific SCARED subscale scores rather than total score, see Tables S6–S10. Outliers were excluded from all between‐subjects analyses if they were >3 standard deviations from the sample mean on the variable being tested, and all predictors were mean centered prior to the computation of interaction terms. Simple slopes from interactions were probed with Johnson‐Neyman tests (Johnson & Neyman, 1936) with robust standard error estimation using the “sim_slopes” function as part of the R package “interactions” (Long, 2019).

In total, 55 participants were excluded from ERP regression analyses for the following reasons: no BI data (*n* = 3), no EEG that passed initial quality checks (*n* = 30), too few artifact‐free trials for reliable ERP signal for one or more components (*n* = 3), missing questionnaires (*n* = 11), and outlier on one or more variables of interest (*n* = 8). The final sample consisted of 112 adolescents. There were no significant differences between participants included versus excluded in analyses in terms of age, sex, highest level of maternal education, BI, anxiety scores, or ERP amplitude; however, African American participants were more likely to be excluded than Caucasian participants (see Table S11; for regression results controlling for these demographic variables, see Table S12). For comparison to traditional regression models, path models using robust maximum likelihood estimation, which allows for the inclusion of all 167 participants, were also tested (see Tables S13 and S14).

## RESULTS

### Task behavior

#### Within‐subjects

Descriptive statistics and bivariate correlations for key variables of interest are presented in Table 1. A one‐way analysis of variance (ANOVA) revealed a significant within‐subjects effect of trial type on correct‐trial probe RT (*F*[3, 654] = 149, *η* ^2^ = 0.41, *p* < 0.001). Post‐hoc tests revealed that BX and BY were the only two trial types that did not differ from each other in terms of RT (*p* = 0.589; all other *p*s < 0.001). Probe RTs were fastest during BX and BY trials (BX: *M* = 299 ms, SD = 69 ms; BY: *M* = 299 ms, SD = 69 ms), followed by AX (*M* = 341 ms, SD = 44 ms) and then AY (*M* = 423 ms, SD = 58 ms).

**TABLE 1:** Descriptive statistics and Pearson correlations

Statistic	Mean	SD	1	2	3	4	5	6	7	8	9	10
1. Behavioral inhibition (standardized)	−0.01	0.77										
2. SCARED total anxiety parent report	10.56	8.82	0.15									
3. SCARED total anxiety child report	20.42	11.68	0.04	0.55***								
4. SCARED total anxiety composite (Z‐Scored)	−0.02	0.83	0.09	0.86***	0.88***							
5. *d'* context	3.13	0.60	−0.03	0.12	0.00	0.06						
6. Cue‐Locked P3b A trials (V/m^2^)	1.11E‐06	6.13E‐07	−0.04	0.02	−0.03	−0.02	0.19*					
7. Cue‐Locked P3b B trials (V/m^2^)	1.44E‐06	7.733E‐07	0.04	0.11	0.16	0.17	0.18*	0.83***				
8. Cue‐Locked P3b B‐A difference (V/m^2^)	3.30E‐07	4.05E‐07	0.06	0.08	0.23*	0.23*	0.14	0.07	0.60***			
9. Probe‐Locked N2 AX trials (V/m^2^)	−1.90E‐07	1.55E‐07	−0.07	0.02	0.09	0.04	−0.12	−0.28***	−0.36***	−0.22**		
10. Probe‐locked N2 BX trials (V/m^2^)	−1.53E‐07	1.44E‐07	−0.11	0.09	0.13	0.13	−0.19*	−0.23**	−0.29***	−0.12	0.33***	
11. Probe‐locked N2 AX‐BX difference (V/m^2^)	−3.89E‐08	1.70E‐07	0.00	−0.05	−0.03	−0.07	0.08	−0.01	−0.02	−0.11	0.62***	−0.50***

*Note:* All values reflect exclusion of outliers (see Data Analytic Strategy). Because the N2 is a negative‐going voltage deflection, lower (more negative) scores on N2 measures indicate a larger signal.

*p < 0.05. **p < 0.01. ***p < 0.001.

**FIGURE 1:**
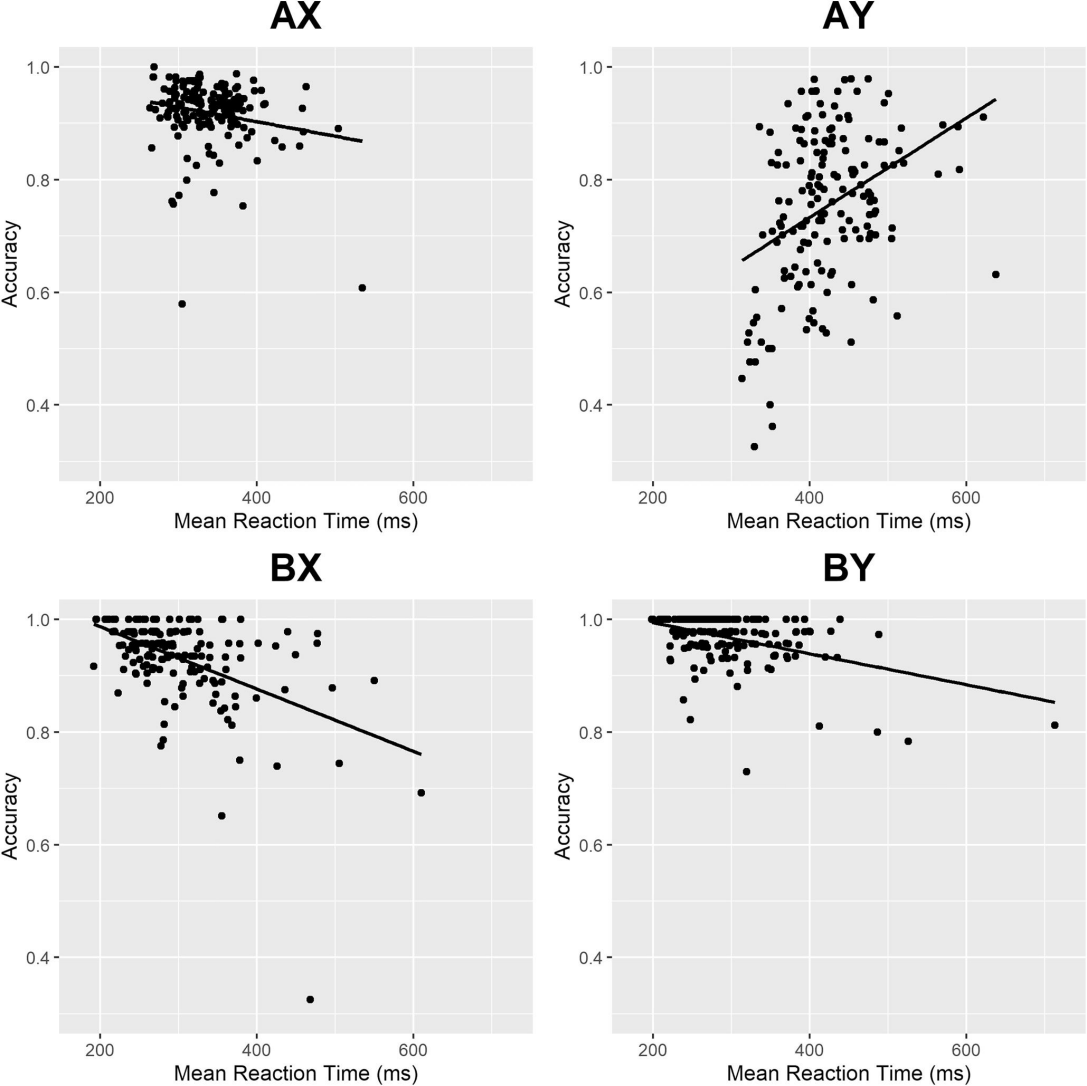
Behavioral performance profiles by trial type. Each data point represents one participant. All four regression lines had slopes significantly different from zero (all *p*s < 0.05). Scatterplots do not reflect the exclusion of outliers

A similar one‐way ANOVA revealed a significant within‐subject effect of trial type on accuracy (*F*[3, 652] = 193, *η* ^2^ = 0.47, *p* < 0.001). Post hoc tests revealed that all trial types were different from each other in terms of accuracy (all *p*s < 0.05). BY trials were most accurate (*M* = 96.7%, SD = 4.5%), followed by BX (*M* = 93.2%, SD = 7.9%), AX (*M* = 91.8%, SD = 5.8%), and AY (*M* = 75.3%, SD = 13.9%). Consistent with past studies of the AX‐CPT in populations predominantly relying on proactive control, AY trials were the slowest and least accurate, whereas BX trials were among the fastest and most accurate (Braver et al., 2009; Cohen et al., 1999; Gonthier, Macnamara, et al., 2016). RT and accuracy profiles by trial type are presented in Figure 1.

#### Between‐subjects

The results of the regression model involving *d'* context appear in Table 2. There were no significant main effects of BI or *d'* context and no significant interaction effect (all *p*s > 0.05).

**TABLE 2:** d’ context regression model predicting total anxiety (Z‐scored)

Predictors	Standardized beta	95% CI	*p*
(Intercept)	0.004	−0.163 – 0.171	0.688
Behavioral inhibition (BI)	0.087	−0.084 – 0.257	0.345
*d'* context	0.082	−0.088 – 0.252	0.346
BI × *d'* context interaction	0.080	−0.094 – 0.253	0.365
Observations	141		
*R* ^2^/*R* ^2^ adjusted	0.020/−0.001		

### Electroencephalography

#### Within‐subjects

**FIGURE 2:**
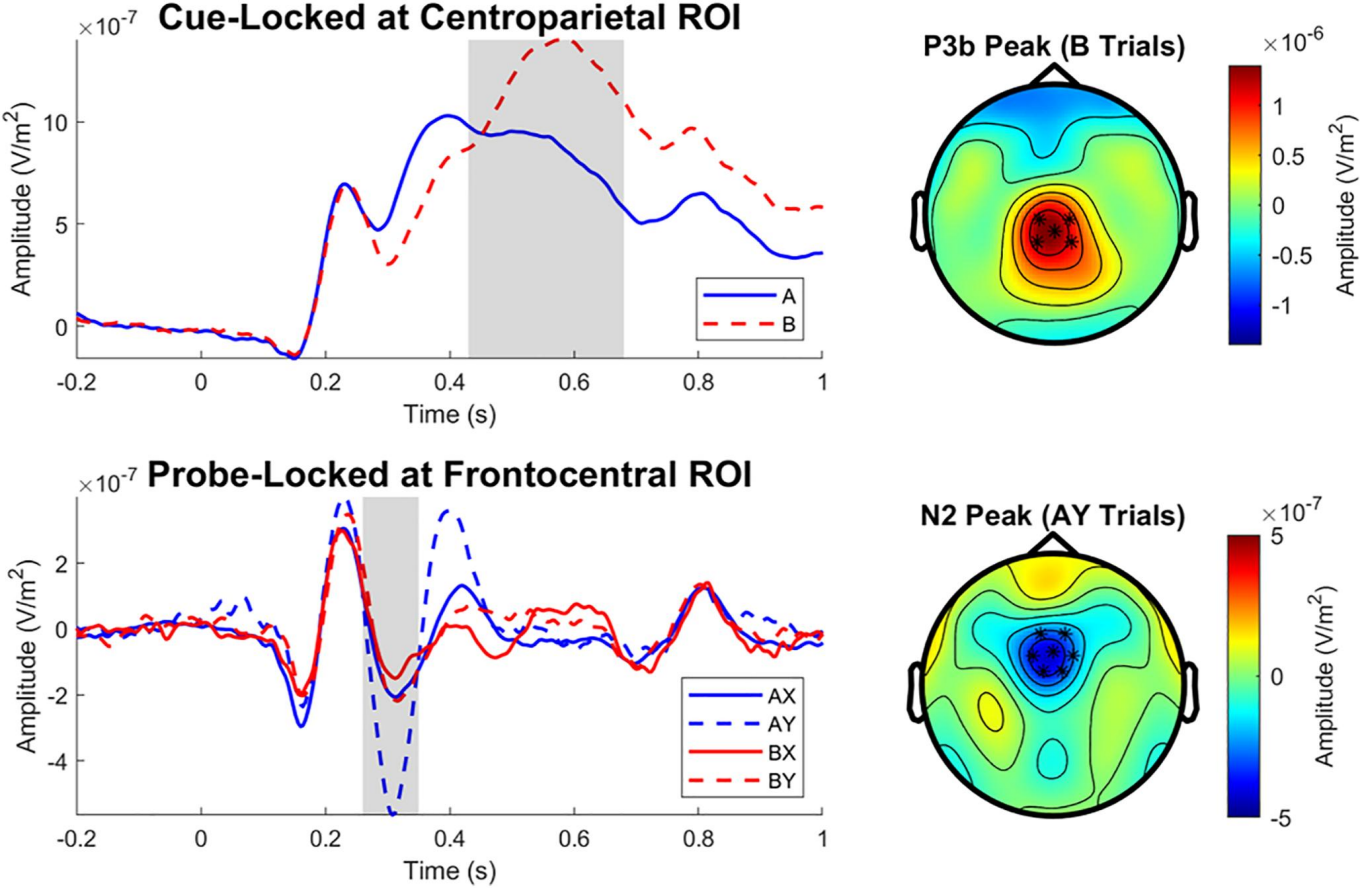
Grand average event‐related potential waveforms. Shaded regions indicate scoring windows. Asterisks on topographic plots indicate locations of sensors included in the given region of interest (ROI). The cue‐locked P3b was significantly more positive following B cues than following A cues (*p* < 0.001). For the probe‐locked N2, all trial types significantly differed from each other (*p*s < 0.05) except for AX and BY (*p* = 0.90)

For grand average cue‐ and probe‐locked ERPs, see Figure 2. Cue‐locked P3b amplitude was significantly more positive following B cues than following A cues (*t*[133] = 9.39, *d* = 0.79, *p* < 0.001). A one‐way ANOVA revealed a significant within‐subjects effect of trial type on probe‐locked N2 amplitude (*F*[3, 538] = 46.90, *η* ^2^ = 0.21, *p* < 0.001). Post hoc tests revealed that all trial types significantly differed from each other in terms of N2 amplitude (*p*s < 0.05) except for AX and BY (*p* = 0.900).

#### Between‐subjects

**FIGURE 3:**
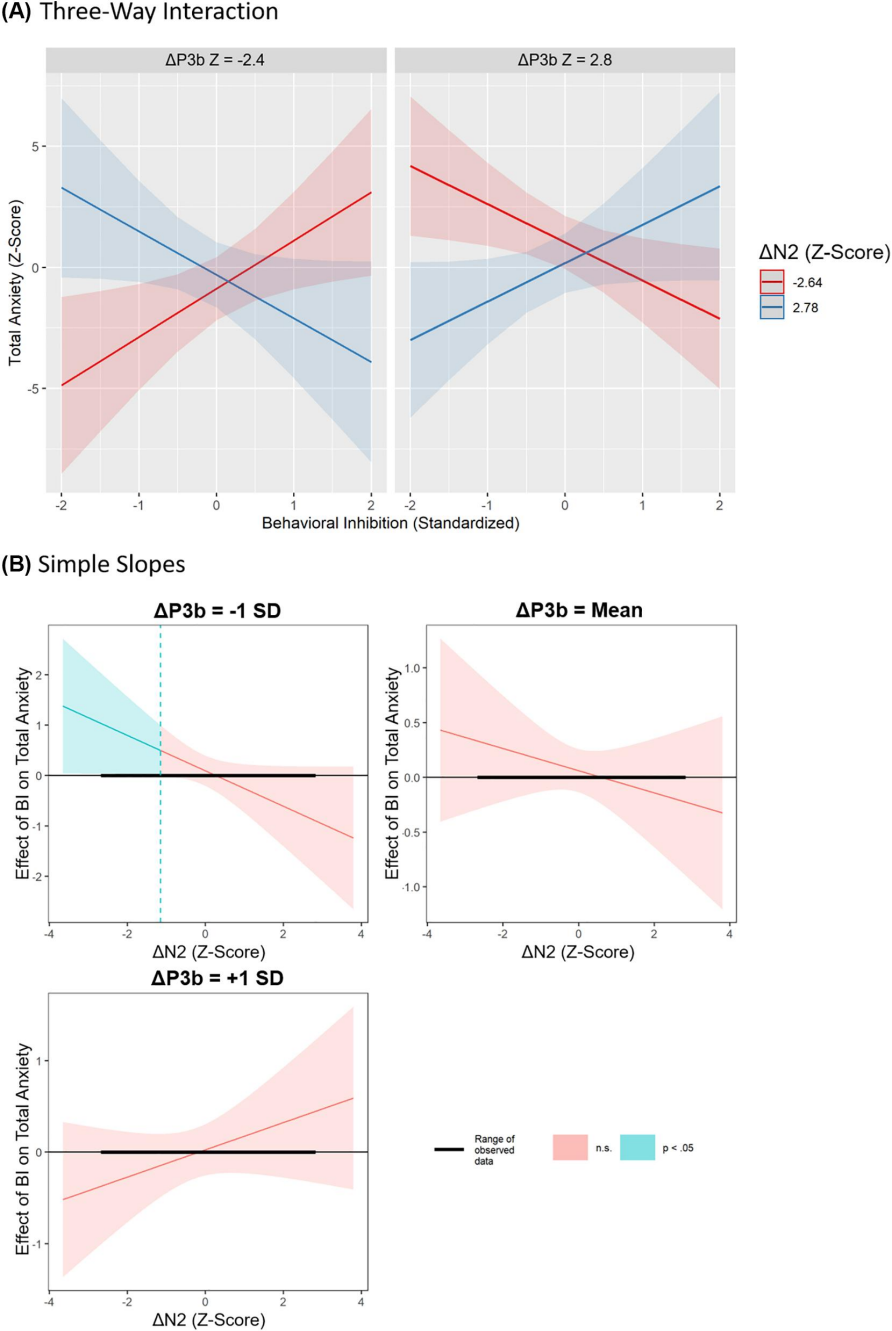
Three‐way interaction and simple slopes. (A) The three‐way interaction between behavioral inhibition (BI), ΔP3b, and ΔN2 (*p*_interaction_ = 0.018), and (B) Johnson–Neyman plots illustrating results of simple slopes analysis, which tested under what conditions the association between BI and anxiety was statistically significant. It revealed that BI is significantly associated with greater anxiety (*p* < 0.05) when ΔP3b is small (i.e., less positive; ΔP3b cutoff: *Z* < −1) and ΔN2 is large (i.e., more negative; ΔN2 cutoff: *Z* < −1.15)

The results of the regression model involving EEG measures are presented in Table 3. There was a significant main effect of ΔP3b such that a larger B‐ minus A‐cue difference (indicating greater use of a proactive strategy) was associated with greater anxiety (*β* = 0.281, *p* = 0.004, $\eta_{p}^{2}$  = 0.057). There was also a significant three‐way interaction between BI, ΔP3b, and ΔN2 (*β* = 0.237, *p* = 0.018, $\eta_{p}^{2}$ = 0.052; see Figure 3, panel A). A Johnson‐Neyman follow‐up test revealed that BI was significantly associated with greater anxiety (*p* < 0.05) specifically when ΔP3b was small (indicating a less proactive strategy; ΔP3b *Z* < −1 [amplitude < −7.52 × 10^−8^ V/m^2^]) and ΔN2 was large (i.e., more negative, indicating a more reactive strategy; ΔN2 *Z* < −1.15 [amplitude < −2.30 × 10^−7^ V/m^2^]; see Figure 3, panel B). See Table S12 for the same model controlling for demographic variables (i.e., age, sex, race/ethnicity, maternal education). Of note, the three‐way interaction between BI, ΔP3b, and ΔN2 remained significant when controlling for participant demographic variables (i.e., age, sex, race/ethnicity, maternal education; see Table S12). However, the main effect of ΔP3b did not. Among these demographic variables, only sex was significantly associated with ERP amplitude. Specifically, females exhibited significantly more positive ΔP3b amplitude than males (*t*[131] = 2.51, *p* = 0.013). Path models revealed effects comparable to those from the traditional regression‐based models (see Tables S13 and S14).

**TABLE 3:** ERP regression model predicting total anxiety (Z‐scored)

Predictors	Standardized Beta	95% CI	*p*
(Intercept)	−0.006	−0.188 – 0.175	0.624
Behavioral inhibition (BI)	0.054	−0.130 – 0.238	0.535
ΔN2	−0.018	−0.222 – 0.186	0.852
**ΔP3b**	**0.281**	**0.093 – 0.469**	**0.004**
BI × ΔN2 interaction	−0.096	−0.308 – 0.116	0.349
BI × ΔP3b interaction	−0.025	−0.224 – 0.173	0.740
ΔN2 × ΔP3b interaction	−0.069	−0.260 – 0.123	0.523
**BI × ΔN2 × ΔP3b interaction**	**0.237**	**0.041 – 0.433**	**0.018**
Observations	112		
*R* ^2^/*R* ^2^ adjusted	0.123/0.064		

*Note:* Bold values indicate statistically significant effects (p < 0.05).

## DISCUSSION

The present study tested whether the relations between early BI and adolescent anxiety vary as a function of reactive and proactive control. We hypothesized that early BI would be associated with greater anxiety, specifically, among adolescents using a strategy characterized by the combination of low proactive control and high reactive control. Because the most relevant past work does not separate proactive and reactive control processes, it was important to use neural measures to separate these two processes. This tested whether past behavioral findings might reflect proactive control, reactive control, or their interplay. The current study used the cue‐locked ΔP3b as a measure of proactive control and the probe‐locked ΔN2 as a measure of reactive control. In line with DMC theory (Braver, 2012), these two measures were not significantly correlated, suggesting they may be relatively independent processes. The study found that the relations between early BI and greater anxiety are only significant among adolescents who use a strategy characterized by low proactive control (i.e., ΔP3b is smaller/less positive, indicating less processing of the cue identity prior to probe onset) and high reactive control (i.e., ΔN2 is larger/more negative, indicating more processing of the cue identify after the probe onset). Thus, proactive and reactive control processes interact to influence the relations amongst BI and anxiety.

It was noteworthy that we did not replicate the 13‐year behavioral findings (i.e., *d*' context moderating the BI‐anxiety association) at this 15‐year assessment. One possible explanation for this involves the normative development of proactive control. Youth at 15 years had numerically higher *d*' context scores than at 13 years (15‐year *M* = 3.13, 13‐year *M* = 2.00; see Troller‐Renfree, Buzzell, Pine, et al., 2019), reflecting a greater reliance on proactive control strategies with age. This is in line with past work showing that proactive control continues to grow in efficiency throughout adolescence and young adulthood (Chevalier et al., 2015). Thus, it could be that by age 15, many adolescents had reached adult or near‐adult levels of proactive control, resulting in a ceiling effect. Such development of cognitive control may relate to the increased use of adaptive and reduced use of maladaptive emotion regulation strategies observed from early adolescence to adulthood (Cracco et al., 2017). Another possible explanation for the d' context change, however, is that the AX‐CPT administered at age 15 had shorter inter‐stimulus intervals due to being adapted for EEG. This may have made it easier to use proactive strategies than in the 13‐year version (i.e., it may have been easier to maintain the cue identity in working memory over the shorter intervals at 15 years). If this is the case, it, too, could partly explain the higher d' context scores at age 15. Regardless, that the ERP measures still significantly moderated the BI‐anxiety relation despite the lack of behavioral differences may highlight the value of such neural measures.

The present findings support an emerging view of BI's neurophysiological profile (Fox et al., 2021). According to this view, BI is associated with heightened detection of salient stimuli (e.g., threatening faces). Nevertheless, some children with BI learn to regulate their responses to novelty or unfamiliarity over time via increased proactive control (Buzzell et al., 2018; Henderson et al., 2015; Henderson & Wilson, 2017). This increase in the deployment of proactive control helps the child recover their goal‐oriented attention and reduces the length of time that attention is shifted toward a salient stimulus when it occurs, thereby ameliorating BI‐related risk for anxiety. In contrast to proactive control, reactive control *maintains* attention toward the salient stimulus and thus, may increase risk for anxiety. Reactive control may help resolve conflict or support quick and reflexive corrections to behavior. Yet, an overabundance of reactive control may contribute to anxious freezing behavior, such as selective mutism, which has been theorized to stem from (reactive) inhibitory *over*control (Muris et al., 2016; Wong, 2010). When operating in conjunction, however, proactive and reactive control support the child's ability to fluidly respond to salient stimuli in goal‐directed contexts.

In addition to the BI pathway described above, results revealed that adolescents with a more positive ΔP3b, which we interpreted as indicating a more proactive strategy, tended to have greater anxiety symptoms. Importantly, however, this effect was no longer significant when controlling for demographic variables. This may have been partly because females had significantly greater anxiety and larger ΔP3bs than males, suggesting that females tended to rely more on proactive control than males. Overall, that the main effect of ΔP3b on anxiety appeared to have been better explained by participant demographics, coupled with the fact that we did not have any specific hypotheses regarding such a main effect, indicates that the direct relation between ΔP3b and anxiety should be interpreted with caution and requires further study.

The present findings may have implications for anxiety intervention efforts. Proactive control may protect youth with BI against elevated anxiety whereas reactive control may increase anxiety risk. As a result, assessments of proactive and reactive control may identify children with BI facing particularly elevated anxiety risk. Such individuals may benefit from existing evidence‐based psychosocial interventions designed for BI youth, such as The Turtle Program (Chronis‐Tuscano et al., 2015) or Cool Little Kids (Rapee et al., 2005). It may even be that these effective treatments, which primarily target parenting strategies, work in part by helping kids develop their self‐regulatory skills (including cognitive control). Targeting cognitive control directly may also be a promising intervention approach for this subgroup of children. Interventions targeting salience detection (e.g., attention bias modification) (MacLeod & Mathews, 2012) may be appropriate for many anxious youth; however, children with BI may respond better to an intervention targeting their precise cognitive control risk factors. For these children, such interventions might seek to enhance proactive control and/or reduce reactive control. These interventions could be particularly important given that heightened salience detection is a core feature of BI (Fox et al., 2005; Kagan et al., 1984). As such, heightened salience detection may be less malleable than cognitive control for children with BI. Preliminary evidence suggests that proactive‐control‐related skills can be enhanced via training (Gonthier, Macnamara, et al., 2016; Li et al., 2018) and that these enhancements may reduce anxiety (Beloe & Derakshan, 2020; Pan et al., 2020). Such trainings may be especially indicated for youth with a history of BI, perhaps either as a standalone intervention or in conjunction with existing psychosocial treatments.

Critically, however, the present study does not allow for causal inferences in the associations between cognitive control processes and anxiety, in part due to the lack of temporal separation (i.e., with the exception of BI, which was assessed during toddlerhood, all other measures were assessed at the same 15‐year time point) but also because this study was necessarily observational in nature (i.e., random assignment was not possible in this context). Moreover, the present longitudinal sample was oversampled for extreme levels of motor and positive or negative reactivity during toddlerhood (Hane et al., 2008), possibly limiting generalizability; however, this oversampling approach was necessary in order to include a sufficient number of children with BI, which is seen in approximately 10%–15% of young children (Fox et al., 2005). Lastly, it is important to note that although we focused on a Z‐transformed composite of parent‐ and child‐reported total anxiety as the outcome measure, the significant three‐way interaction between BI, ΔP3b, and ΔN2 was significant for child‐reported anxiety but not for parent‐reported anxiety when examined individually; however, previous work has shown that the composite score we used relates to a wider variety of naturalistically observed anxious behaviors than either parent‐ or child‐report alone (Bowers et al., 2020). In any case, in addition to benefitting from early laboratory assessment of early BI (as opposed to relying on retrospective report), the present study was aided by a relatively large sample size which provided sufficient statistical power to detect the interaction between BI, proactive control, and reactive control. Because this was a three‐way interaction with a small‐to‐medium effect size ($\eta_{p}^{2}$  = 0.052), further replication is likely needed.

In summary, the present findings suggest that early BI is associated with elevated anxiety symptoms among adolescents who rely more on reactive control strategies (as indicated by a larger ΔN2) and less on proactive strategies (as indicated by a smaller ΔP3b). This may indicate that, among children with early BI, proactive control is protective against elevated anxiety whereas reactive control increases anxiety risk. More broadly, the present study contributes to a growing body of literature showing that facets of cognitive control can buffer or exacerbate the psychopathology risk associated with relatively stable characteristics such as temperament or personality (for reviews, see Fox et al., 2021; Lonigan et al., 2004). Future work would benefit from understanding what and how factors influence the development of cognitive control and strategies thereof.

## CONFLICT OF INTEREST

The authors have declared that they have no competing or potential conflicts of interest.

## ETHICS STATEMENT

This study's protocol was approved by the institutional review board of the University of Maryland, College Park. Informed consent and assent were obtained at each laboratory assessment, as appropriate.

## AUTHORS CONTRIBUTION

Emilio Valadez: Conceptualization, data curation, formal analysis, investigation, methodology, software, visualization, writing‐original draft, writing‐review & editing. Sonya Troller‐Renfree: Conceptualization, investigation, methodology, project administration, writing‐review & editing. George Buzzell: Conceptualization, investigation, project administration, writing‐review & editing. Heather Henderson: Conceptualization, supervision, writing‐review & editing. Andrea Chronis‐Tuscano: Supervision, writing‐review & editing. Daniel Pine: Conceptualization, funding acquisition, supervision, writing‐review & editing. Nathan Fox: Conceptualization, funding acquisition, supervision, writing‐review & editing.

## Supporting information

Supplementary Material

## Data Availability

The data that support the findings of this study are available from the corresponding author upon reasonable request.
