# Supplementary material for: Behavioral inhibition and dual mechanisms of anxiety risk: Disentangling neural correlates of proactive and reactive control
Source: JCPP Adv. 2021 Jul 2;1(2):e12022. doi: 10.1002/jcv2.12022 (PMC8477434; doi:10.1002/jcv2.12022)
Supplement: Supplementary file 1 — Supplementary Material [file JCV2-1-e12022-s001.docx]

**Supporting Information**

**Behavioral Inhibition and Dual Mechanisms of Anxiety Risk: Disentangling Neural Correlates of Proactive and Reactive Control**

Emilio A. Valadez, Sonya V. Troller-Renfree, George A. Buzzell, Heather A. Henderson, Andrea Chronis-Tuscano, Daniel S. Pine, & Nathan A. Fox

**Appendix S1. Supplementary Methods:**

**Participants**

Infants (n=779; age 4 months) completed a laboratory temperament screening for emotional and motor reactivity towards novel auditory and mobile stimuli. From these, infants with high motor and high positive or high negative reactivity were oversampled to reflect a range of temperamental reactivity that is wider than would be found in a randomly selected community sample. The selected infants (n=291) continued to participate in assessments of cognitive and socio-emotional development throughout childhood and adolescence. Informed consent and assent (as appropriate) were obtained at each assessment, and each visit protocol was approved by the institutional review board of the University of Maryland, College Park.

**Behavioral Inhibition**

BI was assessed at ages 24 and 36 months. Behavioral coding of laboratory assessments (Calkins et al., 1996; Fox et al., 2001) and maternal report of social fear (using the Toddler Behavior Assessment Questionnaire) were standardized and then averaged together to create a BI composite score, based on the assumption that combining data from different informants, contexts, and ages reflects a more comprehensive assessment of the child’s temperament (Lahat et al., 2014; Lamm et al., 2014; Walker et al., 2014). During laboratory observations, children were presented with an unfamiliar person and various novel toys (i.e., truck, robot, inflatable tunnel) during three episodes. Measures of interest were proximity to mother and latency to vocalize, to approach and touch the toys, and to approach stranger. The BI composite score was created based on theory and confirmed through principal components analysis (eigenvalue = 2.04; loadings = .62-.81; Lahat et al., 2014).

**Task Stimuli**

Letter stimuli were presented in boldface 60-point Courier New font on a black background. To make clear the distinction between cues and probes, cues were presented in cyan and probes were presented in white. Each trial began with a center fixation cross, followed by the cue stimulus which was presented for 500 ms. The cue stimulus was followed by a randomized interstimulus interval of 1400-1600 ms (fixation cross). The probe was also presented for 500 ms, with a response window of 1000 ms starting from probe onset. Stimuli were presented on a 17-inch LCD monitor using E-Prime 2.0 Professional (Psychology Software Tools, Inc., Sharpsburg, PA).

**Behavior Data Cleaning**

Consistent with past work, individual trials were excluded from analysis if reaction time (RT) was >3 standard deviations above or below each participant’s mean RT on correct trials (Troller-Renfree et al., 2019), resulting in exclusion of less than 3% of all trials (*M* = 2.92%, *SD* = 0.01%). After excluding outlier trials, accuracy and mean reaction times were computed for each trial type. Consistent with other studies with children, participants needed at least 60% accuracy on BY trials for inclusion in analyses (Troller-Renfree et al., 2019, 2020).

***d’* Context**

*d’* context, a commonly used behavioral index based on signal detection theory, provides a measure of the ability to discriminate between target and nontarget trials as a function of the cue (Cohen et al., 1999). *d’* context scores were computed by comparing correct responses on AX trials (hits) relative to incorrect responses on BX trials (false alarms). A correction was applied in cases where there was a hit rate of 1 (hit rate = 2^–(1/N)^, where N = number of target trials) or a false alarm rate of 0 (false alarm rate = 1-2^-(1/N)^, where N = number of nontarget trials). The distribution of *d’* context scores (skewness -0.05, kurtosis 2.57) was inspected and determined to be normal. Consistent with the broader literature involving *d’* context, higher scores were interpreted to indicate a more proactive style of cognitive control because the participant used the cue information to inform future responses (Cohen et al., 1999; Troller-Renfree et al., 2019, 2020).

**Schedule for Affective Disorders and Schizophrenia for School-Age Children – Present and Lifetime Version (K-SADS-PL)**

Although not included in analyses, participants were also administered the K-SADS-PL (Kaufman et al., 1997) as part of the same 15-year assessment. The K-SADS-PL is a semi-structured diagnostic interview for assessing current and past psychopathology among children and adolescents according to DSM-5 criteria and was administered by an advanced graduate student or doctoral level clinician under the close supervision of a board-certified child and adolescent psychiatrist and a licensed clinical psychologist. The SCARED was used in analyses instead of the K-SADS-PL because it provides a continuous, rather than binary, measure of anxiety. Moreover, due to concerns about high shared variance between SCARED scores and diagnostic status, K-SADS-PL diagnoses were not included in models as covariates (Miller & Chapman, 2001). Nevertheless, diagnostic information from the K-SADS-PL are presented here for descriptive purposes. Of the 167 adolescents who participated in the 15-year AX-CPT, 131 were administered the K-SADS-PL. Of these, 61 (46.6%) met criteria for at least one diagnosis at the time of assessment, with 15 (11.5%) meeting criteria for a mood disorder and 39 (29.8%) meeting criteria for an anxiety disorder. Specific anxiety diagnoses included social anxiety disorder (n=21; 16%), generalized anxiety disorder (n=16; 12.2%), specific phobia (n=15; 11.5%), separation anxiety disorder (n=1; 0.8%), and panic disorder (n=1; 0.8%).

**Electrophysiological Pre-processing**

All pre-processing, including ocular artifact detection and removal, was performed with the Maryland Analysis of Developmental EEG (MADE) pipeline (Debnath et al., 2020), which utilizes MATLAB (The MathWorks, Natick, MA) functions from EEGLAB (Delorme & Makeig, 2004) and its plugins “FASTER” (Nolan et al., 2010), “ADJUST” (Mognon et al., 2011), and “ADJUSTED ADJUST” (Leach et al., 2020). Offline, data were re-referenced to an average reference and band-pass filtered from 0.3 to 50 Hz with a digital FIR filter. Data were segmented separately for each of the four trial types (i.e, AX, AY, BX, BY). Only trials with correct behavioral responses were analyzed. Channels were marked bad if voltage exceeded ±150 µV, and any epochs in which more than 10% of non-ocular channels exceeded this threshold were marked bad; otherwise, bad channels were interpolated via a spherical-spline interpolation. Remaining EEG processing steps were performed with a combination of custom MATLAB scripts and the FieldTrip Toolbox (Oostenveld et al., 2010).

**Event-Related Potential (ERP) Reliability Analysis**

Internal consistency with increasing numbers of trials was estimated for each event-related potential (ERP) component and trial type to determine the minimum numbers of trials needed for reliable voltage estimates (see Figure S1). This was performed in R (version 3.6.2) using the procedure described by Leach et al. (2020) and initially proposed by Towers and Allen (2009). Specifically, Spearman-Brown-corrected split-half correlation coefficients were calculated in steps of two trials from 2 to 20 and in steps of five trials from 20 to 40. For each number of trials, *n*, 10,000 iterations of split-half correlations were computed. For each iteration, *n* trials were randomly selected from all available trials for each participant. The selected trials were then halved by randomly assigning trials to one of two halves. The mean ERP amplitude was then calculated separately for each half, and a Pearson correlation was then obtained using each half across all participants for a given *n*. Lastly, the Spearman-Brown prophecy formula was applied to correct each reliability estimate for test length (i.e. estimate the reliability when the number of trials is doubled, given that reliability estimate was calculated with half the original number of trials). This process generated 10,000 reliability estimates for a given n for each component and trial type. Notably, when iterating across number of trials (from 2 to 40), the number of participants included in the split-half reliability estimates decreased as the number of trials increased. All reliability estimates included a minimum of six participants. For the cue-locked P3b, a minimum of 10 trials for A cues and 6 trials for B cues were needed to reach acceptable reliability (i.e., reliability coefficient ≥ 0.6. For the probe-locked N2, a minimum of 12 trials for AX probes and 18 trials for BX probes were needed to reach acceptable reliability. Participants were excluded from ERP analyses if they had too few artifact-free trials for any of these ERP measures. Mean and range of trial counts for remaining participants were as follows: AX: mean = 135.4, range = 63-167; AY: mean = 30.5, range = 13-44; BX: mean = 36.9, range = 21-47; BY: mean = 38.2, range = 18-48.

**Figure S1**

Internal Consistency Estimates with Increasing Numbers of Trials


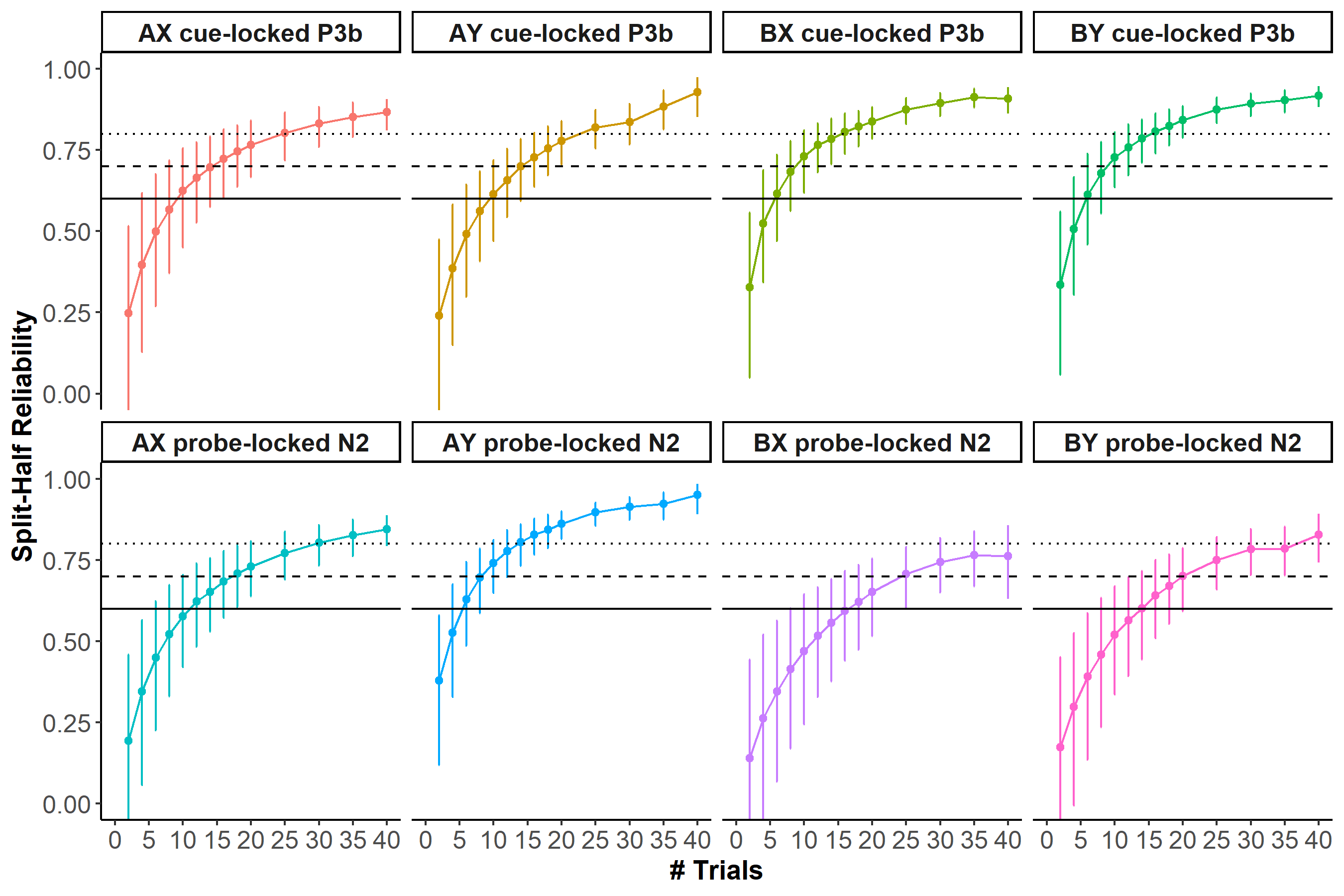


Note. Horizontal lines indicate .6, .7, and .8 reliability. Estimates and 95% confidence intervals were generated from 10,000 randomizations.

***Additional Results***

Table S1

*d’ Context Regression Model Predicting Parent-Reported Total Anxiety (Z-Scored)*

| *Predictors* | *Standardized Beta* | *95% CI* | *p* |
| --- | --- | --- | --- |
| (Intercept) | 0.001 | -0.156 – 0.158 | .428 |
| Behavioral Inhibition (BI) | 0.135 | -0.026 – 0.297 | .107 |
| *d'* Context | 0.126 | -0.033 – 0.285 | .120 |
| BI * *d'* Context interaction | 0.054 | -0.108 – 0.215 | .513 |
| Observations | 155 |  |  |
| R^2^ / R^2^ adjusted | .038 / .019 | |  |

Table S2

*d’ Context Regression Model Predicting Child-Reported Total Anxiety (Z-Scored)*

| *Predictors* | *Standardized Beta* | *95% CI* | *p* |
| --- | --- | --- | --- |
| (Intercept) | 0.003 | -0.163 – 0.169 | .990 |
| Behavioral Inhibition (BI) | 0.039 | -0.131 – 0.209 | .673 |
| *d'* Context | 0.033 | -0.136 – 0.203 | .707 |
| BI * *d'* Context interaction | 0.062 | -0.111 – 0.235 | .480 |
| Observations | 144 |  |  |
| R^2^ / R^2^ adjusted | .007 / -.015 | |  |

Table S3

*ERP Regression Model Predicting Parent-Reported Total Anxiety (Z-Scored)*

| *Predictors* | *Standardized Beta* | *95% CI* | *p* |
| --- | --- | --- | --- |
| (Intercept) | -0.007 | -0.184 – 0.171 | .494 |
| Behavioral Inhibition (BI) | 0.110 | -0.069 – 0.290 | .224 |
| ΔN2 | -0.002 | -0.200 – 0.197 | .985 |
| ΔP3b | 0.077 | -0.106 – 0.259 | .385 |
| BI * ΔN2 interaction | 0.047 | -0.158 – 0.252 | .657 |
| BI * ΔP3b interaction | -0.141 | -0.332 – 0.049 | .135 |
| ΔN2 * ΔP3b interaction | -0.174 | -0.358 – 0.011 | .067 |
| BI * ΔN2 * ΔP3b interaction | 0.065 | -0.119 – 0.250 | .486 |
| Observations | 123 |  |  |
| R^2^ / R^2^ adjusted | .077 / .021 | |  |

Table S4

*ERP Regression Model Predicting Child-Reported Total Anxiety (Z-Scored)*

| *Predictors* | *Standardized Beta* | *95% CI* | *p* |
| --- | --- | --- | --- |
| (Intercept) | -0.002 | -0.185 – 0.181 | .939 |
| Behavioral Inhibition (BI) | 0.030 | -0.155 – 0.215 | .713 |
| ΔN2 | 0.033 | -0.171 – 0.238 | .757 |
| **ΔP3b** | **0.277** | **0.089 – 0.465** | **.004** |
| BI * ΔN2 interaction | -0.106 | -0.318 – 0.107 | .317 |
| BI * ΔP3b interaction | -0.043 | -0.239 – 0.154 | .617 |
| ΔN2 * ΔP3b interaction | -0.038 | -0.228 – 0.153 | .720 |
| **BI * ΔN2 * ΔP3b interaction** | **0.194** | **0.001 – 0.386** | **.049** |
| Observations | 114 |  |  |
| R^2^ / R^2^ adjusted | .098 / .039 | |  |

Table S5

*ERP Regression Model Predicting Total Anxiety (Z-Scored) Using Residualized ERP Difference Scores*

| *Predictors* | *Standardized Beta* | *95% CI* | *p* |
| --- | --- | --- | --- |
| (Intercept) | 0.011 | -0.175 – 0.196 | .919 |
| Behavioral Inhibition (BI) | 0.046 | -0.145 – 0.237 | .634 |
| ΔN2 | 0.011 | -0.193 – 0.216 | .914 |
| **ΔP3b** | **0.297** | **0.100 – 0.494** | **.004** |
| BI * ΔN2 interaction | -0.004 | -0.211 – 0.202 | .965 |
| BI * ΔP3b interaction | 0.006 | -0.202 – 0.215 | .938 |
| ΔN2 * ΔP3b interaction | 0.008 | -0.221 – 0.237 | .917 |
| BI * ΔN2 * ΔP3b interaction | 0.166 | -0.031 – 0.363 | .098 |
| Observations | 114 |  |  |
| R^2^ / R^2^ adjusted | .090 / .030 | |  |

*Note.* ΔP3b reflects the residual from regressing the B-cue P3b (dependent variable) on the A-cue P3b (explanatory variable). ΔN2 reflects the residual from regressing the AX-probe N2 (dependent variable) on the BX-probe N2 (explanatory variable).

Table S6

*ERP Regression Model Predicting Panic Symptoms (Z-Scored)*

| *Predictors* | *Standardized Beta* | *95% CI* | *p* |
| --- | --- | --- | --- |
| **(Intercept)** | **-0.008** | **-0.194 – 0.178** | **.040** |
| Behavioral Inhibition (BI) | -0.020 | -0.208 – 0.168 | .844 |
| ΔN2 | -0.015 | -0.223 – 0.194 | .923 |
| **ΔP3b** | **0.261** | **0.070 – 0.453** | **.007** |
| BI * ΔN2 interaction | 0.073 | -0.146 – 0.293 | .542 |
| BI * ΔP3b interaction | -0.127 | -0.328 – 0.074 | .191 |
| ΔN2 * ΔP3b interaction | -0.106 | -0.301 – 0.089 | .289 |
| BI * ΔN2 * ΔP3b interaction | 0.129 | -0.072 – 0.329 | .207 |
| Observations | 107 |  |  |
| R^2^ / R^2^ adjusted | .129 / .068 | |  |

Table S7

*ERP Regression Model Predicting Social Phobia Symptoms (Z-Scored)*

| *Predictors* | *Standardized Beta* | *95% CI* | *p* |
| --- | --- | --- | --- |
| (Intercept) | -0.008 | -0.192 – 0.176 | .696 |
| **Behavioral Inhibition (BI)** | **0.225** | **0.039 – 0.411** | **.017** |
| ΔN2 | 0.124 | -0.082 – 0.330 | .238 |
| ΔP3b | 0.138 | -0.051 – 0.327 | .142 |
| BI * ΔN2 interaction | -0.085 | -0.299 – 0.129 | .429 |
| BI * ΔP3b interaction | 0.006 | -0.191 – 0.204 | .972 |
| ΔN2 * ΔP3b interaction | -0.097 | -0.288 – 0.095 | .326 |
| BI * ΔN2 * ΔP3b interaction | 0.084 | -0.110 – 0.277 | .393 |
| Observations | 114 |  |  |
| R^2^ / R^2^ adjusted | .086 / .026 | |  |

Table S8

*ERP Regression Model Predicting Generalized Anxiety Symptoms (Z-Scored)*

| *Predictors* | *Standardized Beta* | *95% CI* | *p* |
| --- | --- | --- | --- |
| (Intercept) | -0.004 | -0.188 – 0.181 | .757 |
| Behavioral Inhibition (BI) | 0.076 | -0.110 – 0.262 | .423 |
| ΔN2 | -0.080 | -0.286 – 0.126 | .452 |
| **ΔP3b** | **0.195** | **0.006 – 0.385** | **.042** |
| BI * ΔN2 interaction | 0.058 | -0.157 – 0.273 | .605 |
| BI * ΔP3b interaction | -0.075 | -0.274 – 0.123 | .426 |
| ΔN2 * ΔP3b interaction | -0.069 | -0.261 – 0.124 | .491 |
| BI * ΔN2 * ΔP3b interaction | 0.137 | -0.059 – 0.332 | .168 |
| Observations | 113 |  |  |
| R^2^ / R^2^ adjusted | .092 / .032 | |  |

Table S9

*ERP Regression Model Predicting Separation Anxiety Symptoms (Z-Scored)*

| *Predictors* | *Standardized Beta* | *95% CI* | *p* |
| --- | --- | --- | --- |
| (Intercept) | -0.001 | -0.194 – 0.191 | .142 |
| Behavioral Inhibition (BI) | 0.110 | -0.084 – 0.304 | .231 |
| ΔN2 | -0.051 | -0.264 – 0.161 | .643 |
| ΔP3b | 0.069 | -0.144 – 0.281 | .511 |
| BI * ΔN2 interaction | -0.167 | -0.387 – 0.053 | .137 |
| BI * ΔP3b interaction | -0.027 | -0.245 – 0.192 | .784 |
| ΔN2 * ΔP3b interaction | -0.042 | -0.258 – 0.174 | .690 |
| BI * ΔN2 * ΔP3b interaction | 0.074 | -0.155 – 0.303 | .522 |
| Observations | 109 |  |  |
| R^2^ / R^2^ adjusted | .050 / .016 | |  |

Table S10

*ERP Regression Model Predicting School Phobia Symptoms (Z-Scored)*

| *Predictors* | *Standardized Beta* | *95% CI* | *p* |
| --- | --- | --- | --- |
| (Intercept) | -0.005 | -0.193 – 0.183 | .057 |
| Behavioral Inhibition (BI) | 0.004 | -0.186 – 0.194 | .882 |
| ΔN2 | 0.165 | -0.057 – 0.387 | .140 |
| ΔP3b | 0.156 | -0.038 – 0.349 | .091 |
| BI * ΔN2 interaction | -0.189 | -0.424 – 0.046 | .114 |
| **BI * ΔP3b interaction** | **-0.225** | **-0.428 – -0.021** | **.024** |
| ΔN2 * ΔP3b interaction | -0.148 | -0.339 – 0.044 | .126 |
| BI * ΔN2 * ΔP3b interaction | 0.134 | -0.061 – 0.329 | .175 |
| Observations | 107 |  |  |
| R^2^ / R^2^ adjusted | .110 / .047 | |  |

Table S11

*Comparison of Participants Included Versus Excluded from ERP Analyses*

|  | Included (n = 112) | |  | Excluded (n = 55) | | *p*-value |
| --- | --- | --- | --- | --- | --- | --- |
| Variable | *M/n* | *SD/%* |  | *M/n* | *SD/%* |  |
| Sex (male) | 50 | 44.6 |  | 23 | 41.8 | .74 |
| Age (years) | 15.4 | 0.6 |  | 15.5 | 0.7 | .19 |
| Race/Ethnicity* |  |  |  |  |  | .016 |
| African American | 11 | 9.8 |  | 17 | 30.9 | - |
| Asian | 4 | 3.6 |  | 1 | 1.8 | - |
| Caucasian | 85 | 75.9 |  | 34 | 61.8 | - |
| Hispanic | 8 | 7.1 |  | 2 | 3.6 | - |
| Other | 4 | 3.6 |  | 1 | 1.8 | - |
| Highest Maternal Education |  |  |  |  |  | .58 |
| High school diploma | 20 | 17.9 |  | 9 | 16.7 | - |
| 4-year college degree | 52 | 46.4 |  | 20 | 37.0 | - |
| Postgraduate degree | 36 | 32.1 |  | 22 | 40.7 | - |
| Other | 4 | 3.6 |  | 3 | 5.6 | - |
| Behavioral Inhibition (Standardized) | 0.0 | 0.8 |  | 0.0 | 0.7 | .85 |
| SCARED Parent Report | 10.6 | 9.0 |  | 10.5 | 8.6 | .94 |
| SCARED Child Report | 20.2 | 11.6 |  | 21.0 | 12.1 | .73 |
| SCARED Total Anxiety Composite (Z-Scored) | 0.0 | 0.8 |  | 0.0 | 0.8 | .87 |
| Cue-Locked P3b B-A Difference (V/m2) | 3.4E-07 | 4.1E-07 |  | 2.8E-07 | 3.8E-07 | .52 |
| Probe-Locked N2 AX-BX Difference (V/m2) | -3.3E-08 | 1.7E-07 |  | -7.2E-08 | 1.5E-07 | .36 |

*Note.* All *p*-values reflect independent samples T tests (for continuous variables) or Fisher’s Exact Test (for categorical variables). *Follow-up pairwise tests revealed that African American participants were significantly more likely to be excluded than Caucasian participants (*p*_corrected_ = .020).

Table S12

*ERP Regression Model Predicting Total Anxiety (Z-Scored) Controlling for Demographics*

| *Predictors* | *Standardized Beta* | *95% CI* | *p* |
| --- | --- | --- | --- |
| (Intercept) | 0.008 | -0.163 – 0.178 | .325 |
| Age (years) | -0.067 | -0.245 – 0.110 | .453 |
| **Sex** | **-0.347** | **-0.534 – -0.160** | **<.001** |
| Race/Ethnicity | 0.077 | -0.102 – 0.256 | .397 |
| **Maternal Education** | **-0.271** | **-0.454 – -0.088** | **.004** |
| Behavioral Inhibition (BI) | 0.020 | -0.155 – 0.196 | .814 |
| ΔN2 | 0.038 | -0.154 – 0.230 | .692 |
| ΔP3b | 0.163 | -0.031 – 0.357 | .092 |
| BI * ΔN2 interaction | -0.022 | -0.222 – 0.178 | .792 |
| BI * ΔP3b interaction | 0.037 | -0.158 – 0.231 | .802 |
| ΔN2 * ΔP3b interaction | -0.056 | -0.256 – 0.144 | .619 |
| **BI * ΔN2 * ΔP3b interaction** | **0.219** | **0.004 – 0.433** | **.046** |
| Observations | 108 |  |  |
| R^2^ / R^2^ adjusted | .290 / .209 | |  |

*Note.* Sex was dummy coded as female = 0 and male = 1; thus, a negative beta indicates that female participants had greater levels of anxiety than male participants. Maternal education was coded on an ordinal scale where high school diploma = 0, college degree = 1, and post-graduate degree = 2. Lastly, due to small cell sizes for specific racial/ethnic groups (i.e., n < 5), race/ethnicity was collapsed and dummy coded as Non-Caucasian = 0 and Caucasian = 1. Of the four demographic variables included in the model, only sex was significantly associated with ERP amplitude. Specifically, female participants exhibited significantly greater ΔP3b amplitude than male participants (*t*(131) = 2.51, *p* = .013).

Table S13

*ERP Path Model Predicting Total Anxiety (Z-Scored)*

| *Predictors* | *Estimate* | *SE* | *Z* | *p* |
| --- | --- | --- | --- | --- |
| Behavioral Inhibition (BI) | 0.146 | 0.094 | 1.544 | .123 |
| ΔN2 | -0.011 | 0.080 | -0.139 | .890 |
| **ΔP3b** | **0.187** | **0.066** | **2.847** | **.004** |
| BI * ΔN2 interaction | -0.016 | 0.115 | -0.141 | .888 |
| BI * ΔP3b interaction | -0.115 | 0.085 | -1.346 | .178 |
| ΔN2 * ΔP3b interaction | -0.087 | 0.069 | -1.273 | .203 |
| BI * ΔN2 * ΔP3b interaction | 0.165 | 0.084 | 1.954 | .051 |
| Observations | 167 |  |  |  |
| R^2^ | .096 |  |  |  |

*Note.* Model tested using the R package “lavaan” (Rosseel, 2012). Due to missing data and potential departures from multivariate normality, model was estimated using a robust maximum likelihood estimator (MLR; Yuan & Bentler, 2000).

Table S14

*ERP Path Model Predicting Total Anxiety (Z-Scored) Controlling for Demographics*

| *Predictors* | *Estimate* | *SE* | *Z* | *p* |
| --- | --- | --- | --- | --- |
| Age (years) | 0.048 | 0.109 | 0.444 | .657 |
| **Sex** | **-0.599** | **0.134** | **-4.464** | **< .001** |
| Race/Ethnicity | 0.121 | 0.138 | 0.876 | .381 |
| **Maternal Education** | **-0.297** | **0.087** | **-3.405** | **.001** |
| Behavioral Inhibition (BI) | 0.120 | 0.085 | 1.415 | .157 |
| ΔN2 | 0.025 | 0.071 | 0.350 | .726 |
| ΔP3b | 0.115 | 0.070 | 1.654 | .098 |
| BI * ΔN2 interaction | 0.065 | 0.101 | 0.649 | .516 |
| BI * ΔP3b interaction | -0.062 | 0.083 | -0.752 | .452 |
| ΔN2 * ΔP3b interaction | -0.089 | 0.058 | -1.531 | .126 |
| **BI * ΔN2 * ΔP3b interaction** | **0.177** | **0.082** | **2.145** | **.032** |
| Observations | 167 |  |  |  |
| R^2^ | .270 |  |  |  |

*Note.* Model tested using the R package “lavaan” (Rosseel, 2012). Due to missing data and potential departures from multivariate normality, model was estimated using a robust maximum likelihood estimator (MLR; Yuan & Bentler, 2000). All demographic variables were coded in the same way as in Supplementary Table S12.

**Supporting Information References**

Cohen, J. D., Barch, D. M., Carter, C., & Servan-Schreiber, D. (1999). Context-processing deficits in schizophrenia: Converging evidence from three theoretically motivated cognitive tasks. *J Abnorm Psychol*, *108*(1), 120–133. https://doi.org/10.1037//0021-843x.108.1.120

Debnath, R., Buzzell, G. A., Morales, S., Bowers, M. E., Leach, S. C., & Fox, N. A. (2020). The Maryland analysis of developmental EEG (MADE) pipeline. *Psychophysiology*, *57*(6), e13580. https://doi.org/10.1111/psyp.13580

Delorme, A., & Makeig, S. (2004). EEGLAB: An open source toolbox for analysis of single-trial EEG dynamics including independent component analysis. *Journal of Neuroscience Methods*, *134*(1), 9–21. https://doi.org/10.1016/j.jneumeth.2003.10.009

Kaufman, J., Birmaher, B., Brent, D., Rao, U., Flynn, C., Moreci, P., Williamson, D., & Ryan, N. (1997). Schedule for Affective Disorders and Schizophrenia for School-Age Children-Present and Lifetime Version (K-SADS-PL): Initial Reliability and Validity Data. *Journal of the American Academy of Child & Adolescent Psychiatry*, *36*(7), 980–988. https://doi.org/10.1097/00004583-199707000-00021

Leach, S. C., Morales, S., Bowers, M. E., Buzzell, G. A., Debnath, R., Beall, D., & Fox, N. A. (2020). Adjusting ADJUST: Optimizing the ADJUST algorithm for pediatric data using geodesic nets. *Psychophysiology*, *57*(8), e13566.

Miller, G. A., & Chapman, J. P. (2001). Misunderstanding analysis of covariance. *Journal of Abnormal Psychology*, *110*(1), 40–48. https://doi.org/10.1037/0021-843X.110.1.40

Mognon, A., Jovicich, J., Bruzzone, L., & Buiatti, M. (2011). ADJUST: An automatic EEG artifact detector based on the joint use of spatial and temporal features. *Psychophysiology*, *48*(2), 229–240. https://doi.org/10.1111/j.1469-8986.2010.01061.x

Nolan, H., Whelan, R., & Reilly, R. B. (2010). FASTER: Fully automated statistical thresholding for EEG artifact rejection. *Journal of Neuroscience Methods*, *192*(1), 152–162. https://doi.org/10.1016/j.jneumeth.2010.07.015

Oostenveld, R., Fries, P., Maris, E., & Schoffelen, J.-M. (2010). FieldTrip: Open source software for advanced analysis of MEG, EEG, and invasive electrophysiological data. *Computational Intelligence and Neuroscience*, *2011*, 156869. https://doi.org/10.1155/2011/156869

Rosseel, Y. (2012). lavaan: An R Package for Structural Equation Modeling. *Journal of Statistical Software*, *48*(1), 1–36. https://doi.org/10.18637/jss.v048.i02

Towers, D. N., & Allen, J. J. B. (2009). A Better Estimate of the Internal Consistency Reliability of Frontal EEG Asymmetry Scores. *Psychophysiology*, *46*(1), 132–142. https://doi.org/10.1111/j.1469-8986.2008.00759.x

Troller-Renfree, S. V., Buzzell, G. A., Pine, D. S., Henderson, H. A., & Fox, N. A. (2019). Consequences of not planning ahead: Reduced proactive control moderates longitudinal relations between behavioral inhibition and anxiety. *Journal of the American Academy of Child & Adolescent Psychiatry*, *58*(8), 768-775.e1. https://doi.org/10.1016/j.jaac.2018.06.040

Troller-Renfree, S. V., Buzzell, G., & Fox, N. A. (2020). Changes in working memory influence the transition from reactive to proactive cognitive control during childhood. *Developmental Science*. https://doi.org/10.1111/desc.12959

Yuan, K.-H., & Bentler, P. M. (2000). Robust mean and covariance structure analysis through iteratively reweighted least squares. *Psychometrika*, *65*(1), 43–58. https://doi.org/10.1007/BF02294185
